# Enhancing functional and nutritional properties of wheat-based bakery products using microbial lipases

**DOI:** 10.1016/j.fochx.2025.103435

**Published:** 2025-12-28

**Authors:** Hetsi Goswami, Ashok Kumar Bishoyi, Gaurav Sanghvi

**Affiliations:** a Department of Microbiology, Marwadi University, Rajkot, Gujarat, India

**Keywords:** Lipases, Wheat flour, Bakery products, Dough rheology, Baking enzymes

## Abstract

Lipases are vital enzymes that hydrolyze and modify lipids, significantly enhancing the nutritional, sensory, and functional qualities of wheat-based bakery products. This review summarizes lipase structure, classification, and their mechanistic role in modifying native and added lipids within wheat flour. Reported studies suggest that lipase application led to an increase in the loaf volume by 15–35%, enhanced the dough stability and retention by 20%, and delayed crumb firming by 10–25%, contributing to extending the shelf life for 1–2 days depending on the formulated enzyme dosage. Lipases hydrolyze key lipid classes, producing functional lipid derivatives that improve dough stability, shelf life, antioxidant properties, and the bioavailability of essential nutrients. Additionally, lipases facilitate wheat bran valorization and foster the incorporation of prebiotic and biosurfactant functionalities, supporting clean-label trends. Despite their promise, challenges such as batch-to-batch consistency, consumer safety, and regulatory compliance persist. Overall, this review highlights the potential and current limitations of lipase application, providing direction for their effective integration in bakery innovations focused on quality and health benefits.

## Highlights

• Lipase enhances dough rheology, gas retention, and bread volume via lipid changes.
• Quality improves through enzymatic release of fatty acids, minerals, and antioxidants.
• Lipid restructuring delays staling and extends shelf life, stabilizing crumb softness.
• Supports clean-label by replacing emulsifiers and adding prebiotics, structured lipids.

## Graphical abstract

**Figure:**
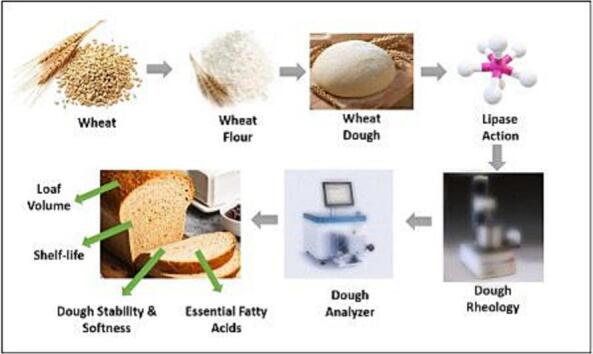

## 1 Introduction

Wheat flour includes 2–3% lipids, which are classed as starch-associated (approx. 40%) and non-starch (approx. 60%) lipids based on where they are located. Bound and free forms of non-starch lipids are categorized based on their solubility in different organic solvents at room temperature and functional interactions with starch and gluten networks. The nonpolar solvents such as hexane, petroleum ether, and ether are used to extract free lipids, and polar solvents like water-saturated butanol are used to remove bound lipids. The granular starch structure contains starch lipids. Usually, they are withdrawn at 90–100 °C using a polar solvent (such as a 90:10 mixture of water and isopropanol or water-saturated butanol) after all non-starch lipids have been eliminated (Abdul Manan, Li, Hsieh, Faubion, & Shi, 2022). Generally, in wheat flour different types of lipids are present, including TAG (triacylglycerol), DGDG (digalactosyldiacylglycerols), NAPE (*N*-acylphosphatidylethanolamines), MGDG (monogalactosyldiacylglycerols), and PC (phosphatidylcholine). Among the diverse lipids present, the non-starch lipids are composed of non-polar lipids (51–63%), galactolipids (22–27%), and phospholipids (13–23%). In contrast to the bound lipid fraction, which is primarily composed of polar lipids, both nonpolar and polar lipids are involved in the free lipid fraction, such as phospholipids and galactolipids. Oleic (10–21%), palmitic (19–26%), and linoleic (50–65%) acids are the main fatty acids found in non-starch lipids. Linoleic (38–48%), palmitic (39–45%), and oleic (8–12%) acids are present in starch lipids, which are primarily phospholipids (85–91%), of which approximately 85% are lysophosphatidylcholines (LPC) (González-Thuillier et al., 2021). Despite lipids low abundance in the wheat flour, these lipids modulate gas cell stabilization, dough rheology, viscoelastic behavior, crumb structure, and staling pattern. Many of these lipids are tightly embedded within starch granules or gluten networks, which limits their availability and surface activity during mixing and fermentation. Their composition also varies widely depending on wheat variety, growing conditions, milling practices, and storage, leading to fluctuations in dough performance, gas retention, and loaf volume. Over time, lipolytic and oxidative changes can further disrupt dough stability by releasing free fatty acids that weaken gas cells, hasten staling, and ultimately diminish the overall quality of the final bread. To further comprehend and overcome these limitations, commercial bakers usually use the synthetic emulsifiers like DATEM, SSL, and glycerol monostearate. These surfactants act like helpers in the dough, maintaining stability for gas cells, improving loaf volume, and making up for the natural shortage of surface-active lipids in wheat flour (Stemler, Hoefflin, & Scherf, 2025). However, use of chemical emulsifiers poses challenges like clean label, regulatory compliance, and shifting of consumer preferences for bio-based functional foods. For the formulation of quality wheat-based bakery products, the hydrolysis of phospholipids and galactolipids is crucial for increasing the final bread volume, according to an examination of lipase-treated wheat lipids (Melis & Delcour, 2020). Mechanistically, the mode of action of these chemical emulsifiers is product specific and cannot replicate lipid restructuring naturally as done by enzymatic hydrolysis. Growing concerns over the use of synthetic emulsifiers have increased interest in identifying the bio-based, clean-label, and sustainable alternative that can naturally regulate the wheat lipid hydrolysis during dough preparation.

Among natural bio-based solutions, hydrolytic enzyme lipase presents a sustainable substitute for chemical emulsifiers. Lipases produce monoglycerides and free fatty acids by breaking down the ester bonds of glycerides and fatty acids at triglyceride sites 1 and 3, increasing the amount of polar lipids in the dough (Melis & Delcour, 2020). It also strengthens the gluten matrix, which increases the volume of the bread loaf. Additionally, glycolipids are more crucial in baking than phospholipids, according to a 1976 theory by De Stefanis and Ponte (Zhao & Xu, 2022). The gluten matrix, confined as the bound portion, is composed of the polar lipids. The dough liquor retrieved from lipase-treated dough had higher lipid contents than dough liquor retrieved via control dough, according to research by Melis and Delcour (2020). The liquid film formed due to lipase interaction, which is a component of dough liquor, is thought to be the medium for the integration & proliferation of cells of gas in dough. This implies that the impact on bread volume increases with the amount of lipase-induced surface-active polar lipids in the dough liquor. This clearly suggests the importance of lipase in bakery formulations, as it stabilizes gas cell walls, maintaining dough rheology, elasticity, crumb structure, and shelf life of final baked products.

The application of the lipase enzyme as a dough enhancer is relatively new, and there aren't many studies on its usage. Lipases produce monoglycerides and free fatty acids by breaking down the ester bonds of glycerides and fatty acids at triglyceride sites 1 and 3, increasing the amount of polar lipids in the dough (Melis & Delcour, 2020). Additionally, it strengthened the gluten net, which increased the volume of the bread loaf. The purpose of this review was to investigate the significance of a balanced lipid in baked goods and to provide a technique that completely accounts for the beneficial benefits that lipases have. In order to determine the functionality and technical consequences of various fats, whether endogenously present or enzymatically liberated, lipase application enables the selective manipulation of endogenous wheat lipid without changing other flour ingredients. It was possible to determine the ideal ratio of lipids in bread or cake preparation by utilizing varying lipase levels. Given the functional, nutritional, and technological importance of lipase-driven lipid modification, it is important to understand the structure, specificity, and biochemical behavior of these enzymes to use them effectively in wheat-based bakery products. The following section outlines the key biochemical properties, structural features, and sources of lipases that are most relevant to their roles in food processing.

## 2 Lipases: Biochemical properties and sources

### 2.1 Lipase structure and classification

Lipases, also known as triacylglycerol acyl ester hydrolases, are a class of enzymes that are widely distributed in nature and are classified as carboxylic acid esterases (EC 3.1.1.3). They are members of the serine hydrolase family (Paluzar, Tuncay, & Aydogdu, 2021). The disulfide bond, oxyanion hole, binding pocket, and lid are the main structural elements of lipase. Lipases have a structure that resembles a lid or flap and is crucial for exposing a hydrophobic patch when a substrate is present. It is composed of one or more α helices of different lengths and has two hinge segments on both ends.

The lipase binding pocket is found on the central β sheet and can take the shape of a tunnel, funnel, or hydrophobic fissure that is close to the protein surface. Another crucial element that significantly affects the enzyme's catalytic effectiveness is the oxyanion hole. A negatively charged tetrahedral intermediate is produced during the process of hydrolysis, and fixation of the resulting oxygen ion is obtained via hydrogen bonding, where oxyanion hole residues play a critical part (Akram, Mir, Haq, & Roohi, 2023). Through hydrogen bonding, the oxyanion hole residues are essential in stabilizing this oxygen ion. The nucleophilic serine residue is always adjacent to one of the oxyanion hole residues, whereas the second residue is situated between the α helix and the β3 strand. Furthermore, lipases are cysteine-rich proteins that preserve their structure by containing one to four disulfide linkages. By lowering the protein's entropy, disulfide bridges that develop between cysteine residues greatly enhance conformational stability. ∼21 Using X-ray crystallographic research, a recent study examined the structural changes in cutinases, fungal lipases, and digestive lipases when surfactants or inhibitors are present (Kumar et al., 2023).

**Fig. 1:**
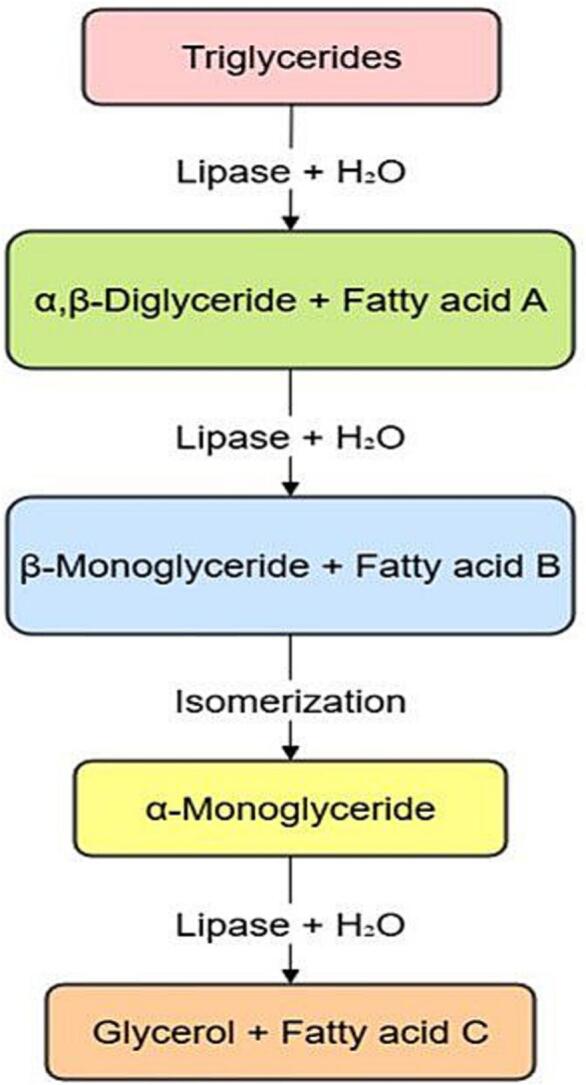
Lipase-mediated hydrolysis of triglycerides illustrating the formation of diacylglycerols, monoacylglycerols, glycerol, and free fatty acids.

**Fig. 2:**
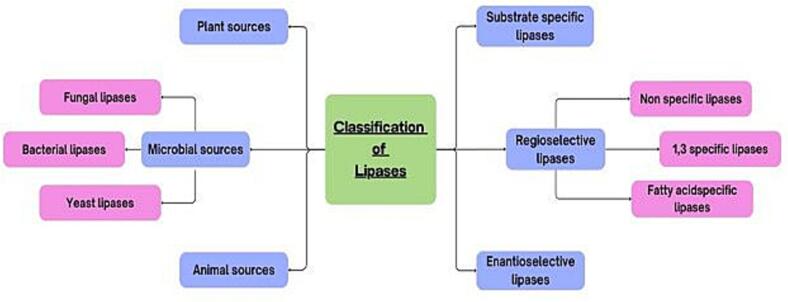
Systematic classification of lipases according to their biological origin and catalytic specificity, including regiospecific and enantioselective subclasses.

The specificity-based lipase classification is covered in this part, while the sources of lipases are covered in the section that follows. Lipases are widely distributed and catalyze a variety of processes. As seen in Fig. 1, lipases can generally be categorized according to both their origins and specificities. The classification of lipases, as shown in the following sections, can provide a clearer understanding of their availability, functionality, and broad range of applications (Fig. 2). When illustrating the commercial uses of lipases, specificity is a crucial factor that allows them to be divided into three main groups: substrate-specific, regioselective, and enantioselective.

### 2.2 Lipase characteristics

These enzymes can hydrolyze triglycerides to produce glycerol, monoacylglycerols (MAGs), diacylglycerols (DAGs), and free fatty acids; conversely, they can use aminolysis, transesterification, and esterification processes to create new products in organic media (Abd Razak, 2022). The catalytic triad of lipases is extremely conserved and consists of histidine, an acidic residue (aspartate/glutamate), and serine as a nucleophile. An electrophilic area known as an oxyanion cavity is formed by a collection of hydrophobic residues that surround the catalytic serine in the active conformation of lipases. Disulphide bridges, which provide stability and are essential for their catalytic activity, are another characteristic of lipases (Gihaz et al., 2020). Additionally, certain lipases contain a structural element known as the “lid” that covers the active site and opens at hydrophobic/hydrophobic interphases. Esterases were classified as lipolytic enzymes without a lid in earlier classifications. However, an alternate classification has been recommended, like CALB (Candida Antarctica Lipase B), which is not identified by the existence of the lid.

### 2.3 Lipase-catalyzed reactions

Microorganisms, plants, and mammals all mass-produce lipases, which are ubiquitous enzymes (Abdelaziz, Abo-Kamar, Elkotb, & Al-Madboly, 2025; Lim, Steiner, & Cridge, 2022). The recombinant manufacturing mechanism is necessary for adoption due to the rising economic interest in these proteins in the food and nutraceutical industries. By employing cell factories for the heterologous manufacture of lipases, the productivity of lipase production bioprocesses has been rising and the cost of enzymes has decreased. Among them, the most often utilized cell factory is *Komogataella phaffi* (*P. pastoris*) (Nieto-Taype et al., 2020). Natural evolution methods, DNA shuffling, protein engineering, site-directed mutagenesis, directed evolution, saturation mutagenesis, and bioinformatics design have been applied to enhance lipases (Hamdan et al., 2021).

## 3 Application of lipases in wheat-based systems

A prior review examined the usage of lipases in wheat-based food systems, like bread & cakes. The food industry may use a variety of lipases, which fall into three broad categories: galactolipases, phospholipases, and triacylglycerol lipases, or the “real” lipases (Reyes-Reyes, Valero Barranco, & Sandoval, 2022). In general, white dough responded better to the ingredients than whole wheat dough. As evidenced by a lessening in the degree of softening and a rise in dough hardness, lipase had a hardening effect on the dough. By reducing dough stickiness, both lipases may improve whole wheat dough's machinability. Lipase activity in dough has been attributed to the production of surface-active chemicals by the hydrolysis of polar and nonpolar lipids. Other mechanisms may possibly be involved, according to the authors. Endogenous lipases may become more active when exposed to exogenous lipases. Both lipase groups release monoglycerides that can attach to gluten proteins and reduce their hydrophobicity, changing the characteristics of dough. The way that gluten and starch interact may be impacted by the binding of gluten by free monoglycerides.

### 3.1 Structured lipids

Structural lipids are essentially re-engineered triacylglycerols where the type and positioning of fatty acids on the glycerol backbone are deliberately modified to achieve targeted nutritional and functional properties. SLs differ in terms of the total amount of saturated and/or unsaturated fats in the total lipid profile. SLs are formed using the method of regiospecific enzymatic catalysis, generally involving lipases that are sn-1 and sn-3 specific, to introduce or exchange fatty acids and maintain or establish desired sn-2 positioning during digestion (Ferreira-Dias, Osório, & Tecelão, 2022; Guo et al., 2020). Due to these structural changes in the position of fatty acids, there is a vast change in the way the body absorbs the fats. Lipases, which perform interesterification, acidolysis, and transesterification, are used to rework natural oils into SLs like low-calorie TAGs, milk fat substitutes that contain sn-2 palmitate, and lipids that work well in baked goods (Reyes-Reyes et al., 2022). Immobilized lipases can also be used to make the process more efficient and consistent and help put the right types of fatty acids in accordance with nutritional goals. Further, acquiring structured lipids allows one to choose the kind of fatty acids to utilize and the location of the molecule to be restructured. Stereospecific enzymes are used in this process to produce new lipids with a stable structure. In an anhydrous reaction medium, lipases catalyze the binding of a fatty acid to a glycerol molecule, while in an aqueous medium, they can hydrolyze a triglyceride. By recycling them in subsequent batches, immobilized biocatalysts have recently reduced the cost of producing structured lipids (Ferreira-Dias et al., 2022). These products, like cocoa butter, low-calorie or enhanced triacylglycerols, and breast milk substitutes, can be produced for nutritional, pharmaceutical, or industrial application using all the knowledge that has been acquired on the topic (Ferreira-Dias et al., 2022; Mota et al., 2020). Therefore, the definition of structured lipids goes beyond just the alteration of levels of saturated and unsaturated fats in total to include an engineered architecture (or construction) of the fatty acid portion of triacylglycerols through the use of enzyme-based positional modification. In many situations, they may not always be able to satisfy nutritional needs even when they are accessible as raw materials. For example, limits on the daily consumption of trans and saturated fatty acids have been raised due to their association with cardiovascular diseases (Astrup et al., 2020; Temkov & Mureșan, 2021). Access to cocoa butter is another obvious example; external factors, including climate change, price fluctuations, and availability, may limit its availability (Kongor, Owusu, & Oduro-Yeboah, 2024). Thus, it makes sense to look for alternatives to deal with these important problems.

Triacylglycerols with fewer calories have lower energy equivalents. These substances are usually utilized to manage other metabolic issues and inadequate absorption of fatty acids. According to Li et al. (2025), they are distinguished by triglycerides (TAGs) of this type that have short- or medium-chain fatty acids in the sn-1,3 positions and a long-chain fatty acid esterified in the sn-2 position. As a result, the absorption of exogenous fatty acids is facilitated, and their metabolism occurs quickly. SLs could be synthesized either through interesterifying oil or TAGs with ethyl or methyl esters, or by acidolyzing an oil/TAG consisting of long-chain fatty acids using one medium-chain fatty acid. Ethyl esters are favored as acyl donors in the lipase-catalyzed synthesis of SLs; methanol presents toxicity hazards in food production (Zhang et al., 2020). In this synthesis approach, *R. oryzae* from regioselective lipase (sn-1,3), immobilized on magnetic nanoparticles, was employed as a catalyst to produce low-calorie triacylglycerols. Cost-effective oils used as raw material were taken from agricultural food surpluses, like olive pomace & spent coffee grounds. This enzyme exhibited higher activity than the commercially available immobilized *T. lanuginosus* lipase, emphasizing its potential for SLs formulated from these oils. Additionally, the enzyme having excellent stability during interesterification & acidolysis of olive pomace oil, the two oils under evaluation showed a predilection for acidolysis. However, due to their fatty acid composition and distribution, SLs can be used to produce products that can partially or completely replace human milk in some situations. These products are designed to improve the consumption of minerals and fat, encourage watery stool, & lessen obstipation in infants (Ferreira-Dias et al., 2022; Jiang, Zou, Chao, & Xu, 2022). An immobilized regioselective lipase is typically used in solvent-free or solvent-containing environments to enzymatically interesterify vegetable, animal, or oil mixes in order to create HMFS (Human Milk Fat Substitute) (Shimane, Ogawa, Yamamoto, & Hara, 2021).

Recently identified lipase from *C. parapsilosi* was employed as a biocatalyst to create HMFS by interesterifying ethyloleate in a solvent-free medium with tripalmitin, offering a novel substitute for commercially available immobilized lipases. Studies employing this method will continue to progress steadily since human milk is a complicated mix of natural lipids.

### 3.2 Bakery products

The creation of novel formulations that adhere to green or less hazardous labeling is prompted by new standards for bakery goods. Lipases have been effectively used to enhance dough processing, strength, volume, structure, and softness in bakery products. They also reduce stickiness and improve both quality and shelf life (Dai & Tyl, 2021; Melis & Delcour, 2020). Since dough is a semi-solid that transforms into a solid cellular sponge when baked, it has a significant impact on the final structure of bread. The lipid portion of ingredients like wheat flour, eggs, or baker's fat significantly contributes to gas incorporation and stabilization, which are vital for producing a foamy and well-aerated yield (Melis & Delcour, 2020). Despite wheat flour's low lipid content, storage duration affects the freshness and quality of bread.

Recent studies have focused on how the lipid fraction of wheat interacts with reformulated flours and added lipids from other sources. Lipases, in particular, are being used to study the effects of both endogenous and exogenous lipids on breadmaking. Lipase action on galactolipids enhances bread volume by hydrolyzing these substrates. For example, flour defatting followed by reconstitution with different lipid fractions helped establish a direct link between specific endogenous lipids and loaf volume enhancement. In parallel, 3 lipases—Lipolase, Lipopan F, and Lecitase Ultra—in addition to the sodium stearoyl lactylate (SSL) surfactant, were evaluated to understand their effect on bread crumb firmness during storage. The surfactants delayed crumb firming through the formation of amylose–lipid insertion complexes, which are supported by the increase in free fatty acids generated during lipase action (Min et al., 2020). Some endogenous wheat lipids mimic surfactant structures, further promoting complex formation and improving texture retention. This enzymatic mechanism also raises regulatory considerations. Residual lipase activity post-baking must be assessed, as European Union clean label regulations require enzymes to be exempt from labeling only if they have no functional effect in the final product (Stemler et al., 2025). Beyond bread, the effect of lipases on cakes has also been studied. By analyzing the lipid composition of various cake formulations before and after lipase treatment, researchers have identified substrate-specific and dose-dependent improvements in baking quality. Lipase-treated formulations showed increased loaf or cake volume, enhanced emulsification, and modified lipid profiles similar to those observed in bread (Stemler et al., 2025).

Lipid composition also varied with cake type and baking status. For example, baked basic cakes showed increased diversity in lipid classes, including phosphatidylcholine and lysophosphatidylcholine. Pound cake exhibited consistent levels of monoacylglycerols and phosphatidylcholine in both batter and baked forms, with lysophosphatidylcholine emerging only after baking. Brioche contained all lipid classes before and after baking, though baking increased the levels of polar lipids such as lysophosphatidylcholine and phosphatidylcholine. These findings enhanced extractability of polar lipids and reduced recovery of nonpolar lipids like triacylglycerols after baking (Melis & Delcour, 2020). Therefore, changes in lipid extractability must be considered when interpreting lipase activity across different bakery matrices.

### 3.3 Lipid extraction

Although lipids constitute only 2.0 to 2.5% of wheat flour by dry weight, they have a significant impact on determining the quality & functionality of cereal-based foods. However, lipid changes during flour maturation have been less explored compared to those of starch and protein. A study by Chen et al. (2024) investigated the lipid profile of freshly milled wheat flour stored at 15 °C, 25 °C, and 40 °C over 60 days. The results showed a continuous increase in free fatty acids, with the greatest increase (50%) occurring at 15 °C. Conjugated trienes peaked at 40 °C, reflecting accelerated lipid peroxidation at higher temperatures. The *p*-anisidine values initially rose and later declined, while total oxidation values became inconsistent after 30 days. These trends suggest that enzymatic activities—especially lipase, lipoxygenase, and catalase—drive lipid oxidation during flour maturation. Such findings are important for optimizing lipid stability and extraction in wheat-based products. To enhance the extraction and profiling of these lipids, Abdul Manan et al. (2022) developed a protease-assisted extraction method using water-saturated butanol. This method significantly increased the lipid yield—from 1.0 ± 0.1% to 1.9 ± 0.3%—and improved phospholipid recovery (5.9 ± 0.8 nmol/g compared to 2.4 ± 1.3 nmol/g in untreated flour). The enhanced extractability offers deeper insight into lipid–protein interactions and better characterization of flour lipids.

Specific protocols for lipid extraction have also been used in dough-based systems. Melis and Delcour (2020) extracted lipid fractions in both free and bound forms from wheat flour, freshly mixed dough, and fermented dough. Each 1.00 g sample was blended with 28 g of acid-washed sand (Sigma-Aldrich, Belgium). For wheat flour, triplicate extractions were conducted, whereas single extractions were used for each dough sample per treatment. All dry lipid extracts were stored at −80 °C in nitrogen-filled, amber-colored vials until further analysis. In the context of composite flours, Olagunju, Ekeogu, and Bamisi (2020) studied breads with 34% wheat flour substituted by acha and pigeon pea flours. They found that lipid extractability and binding behavior varied across mixing, fermentation, and baking stages. Covalent binding of lipids to starch granules not only reduced bread volume but also decreased starch digestibility while enhancing antioxidant capacity and shelf-life stability.

### 3.4 Dough rheology and bread making

Bread samples were prepared at a 10 g scale using a modified straight-dough method, with the exclusion of shortening, malted barley flour, potassium bromate, and ascorbic acid. Using a 10 g pin mixer (National Manufacturing, Lincoln, NE, USA), 10.00 g of flour on a 14.0% moisture basis was combined with 5.7 mL of deionized water, 0.53 g of yeast, 0.15 g of salt, and 0.60 g of sugar for a mixing duration of 4.0 min. By using a Farinograph (Farinograph E, Brabender, Duisburg, Germany), optimal mixing time and water absorption levels were determined. In accordance with AACCI Approved Methods 5421.02 & 5440.02, respectively, mixograph analyses were conducted (Melis & Delcour, 2020). Although lipids are minor constituents of wheat flour, they contribute significantly to bread-making quality. Melis and Delcour (2020) studied that lipase from *Fusarium oxysporum* was used to show that certain lipid classes, especially monogalactosyldiacylglycerols and *N*-acyl phosphatidylethanolamines, improve loaf volume through the formation of lysolipids. However, excessive degradation of lipids led to reduced volume, underscoring the significance of maintaining a balanced lipid profile. Optimal dough gas cell stability relies on preserving lipids that support lamellar mesophase structures while regulating those that favor hexagonal and cubic phase transitions.

Further studies demonstrated that supplementing whole wheat flour with amaranth and acha flours, up to a 30% inclusion level, significantly altered dough rheology and bread-making properties. The composite flours showed modifications in pasting and viscoelastic behavior, reflecting changes in the gluten matrix and starch interactions. These changes enhanced the nutritional value of the bread by expanding protein, fiber, and ash content while reducing the carbohydrate amount. Breads with higher amaranth content also exhibited improved antioxidant capacity and enzyme inhibition activities (α-amylase IC50 ranging from 1.41 to 1.90 mg per mL and α-glucosidase IC50 ranging from 0.83 to 1.39 mg per mL). These breads also had a lower glycemic index between 42 and 51, compared to a glycemic index of 65 for white bread, indicating their potential as functional bakery products (Olagunju, Oluwajuyitan, & Oyeleye, 2021).

To facilitate consistent lipase incorporation into dough formulations and avoid weighing errors due to the insolubility of Lipopan F granulates in the water, a lipase extract was prepared. In a screw cap test tube, 100 mg of Lipopan F and 5.0 mL of deionized water were shaken at 150 rpm for 30 min at room temperature. The mixture was then centrifuged at 4000 *g* for five minutes, followed by filtration of the supernatant. This extract contained the soluble material derived from 20 mg granules of lipase per mL. The supernatant was further diluted with deionized water in ratios varying from 3.80 to 2281. To prepare dough, 5.7 mL of the diluted lipase solution replaced the standard water volume. For instance, a 3.8-fold diluted extract resulted in dough containing material recovered from 30 mg of lipase granulates, corresponding to a concentration of 3000 mg per kg or parts per million. Lipase was incorporated at various levels, varying from 5 to 3000 ppm, depending on the experimental requirement (Melis & Delcour, 2020). The nutritional profile and dough rheology of refined wheat flour can be improved by partially substituting it with quinoa flour at different particle sizes and inclusion levels. Increasing quinoa particle size reduced falling number, water absorption, gas retention, and bread volume. Higher substitution levels led to decreased dough stability, extensibility, and gelatinization characteristics. The optimal addition levels for improved rheological and baking performance in wheat-based bread were 9.14% for large, 10.58% for medium, and 10.24% for small particle sizes (Coţovanu & Mironeasa, 2022). These blends support the development of value-added wheat bread with enhanced functional and nutritional properties.

## 4 Nutritional enhancement through lipase activity

### 4.1 Micronutrients

Wheat grains are a rich source of macronutrients, consisting of proteins, carbohydrates & fats; micronutrients like minerals and phytochemicals; vitamins; Total Dietary Fiber (TDF); and a variety of bioactive compounds. Various colored wheat varieties are valued for their natural pigments, including anthocyanins and carotenoids (Al-Tarifi, Mahmood, Assaw, & Sheikh, 2020). Due to their health benefits and role in disease prevention, pigmented wheat has attracted considerable attention from the food industry and researchers. Pigmented wheat is an abundant source of the vitamin-B group and vitamin E (tocopherol/tocotrienol). Apart from these, it also contains a minute concentration of provitamin A (−carotene), vitamin D (calciferol), and vitamin K (phylloquinone) (Dangi et al., 2023). Iron is a vital component of hemoglobin and essential for overall human development, while zinc is important for brain development and calcium is useful for maintaining healthy bones. & Iron amount in pigmented wheat is around, to, and higher than common wheat, respectively. Wheat kernels and flour consist of increased zinc, while blue-pigmented wheat consists of a higher calcium amount (Dangi et al., 2023). Especially under different planting conditions, all micronutrient amounts of pigmented wheat are not always higher than common wheat.

### 4.2 Bioactive compounds

Bioactive components are essential to human health. Every wheat variety has a number of significant phytochemicals, such as tocols, lignans, sterols, phenolic acids, alkylresorcinols, and folate. Approximately 99% of the Total Phenol Content (TPC) in the bran layer is found in durum wheat bran and bread, which had average gallic acid equivalents of TPC of 9208.52 and 9798.52 mg per kilogram, respectively. Feng et al. (2022) stated that slightly purple pigmented wheat has decreased levels of antioxidant, TPC, and TFC (Total Flavonoid Content), while black wheat has the greatest levels. Black pigmented wheat was found to contain 659.8 μg gallic acid analogues and 318.9 μg analogues of TPC and TFC, respectively, according to the study. The nutritional advantages of the wheat bran layer are highlighted by the fact that the 2 elements are higher in debranned wheat flour and whole wheat flour than in refined flour (*p* < 0.05) (Chen, Mense, Brewer, & Shi, 2024; Guan, Zhang, & Tan, 2023).

The Total Antioxidant Capacity (TAC) in various colored wheat was calculated using two distinct extraction techniques: the Accelerated Solvent Extraction method (ASE) and Solvent Extraction (SE). (Saini et al., 2021) According to their findings, TAC for SE and ASE procedures ranges from over 17.81 to 298.31 ppm & 12.5–282.3 ppm, respectively. Red & amber wheat had the lowest TAC values, while dark purple, blue, and purple wheat had the greatest TAC values. *T. aestivum* Ssp. *spelta*, *T. monococcum* ssp. monococcum, *T. monococcum* ssp. thaoudar, *T. monococcum* ssp. *aegilopoides*, *T. urartu*, & *T. turgidum* ssp. dicoccum are among the wild wheat species that contain a significant amount of anthocyanins, as do several pigmented wheat species, such as *T. monococcum* (einkorn), *T. aestivum* (bread wheat), & *T. durum* (durum wheat).

Table 1 presents a comparative overview of the nutritional and functional enhancements achieved through lipase addition to wheat-based bakery systems. Across multiple studies, lipase-treated matrices demonstrated significant improvements in micronutrient bioavailability, attributable to increased release and assimilation of essential fatty acids and sterols from complex lipids (Saini et al., 2021). Dough rheology was optimized through improved elasticity and gas retention, a direct result of enzymatic interactions between lipid derivatives and gluten proteins (Melis & Delcour, 2020). Moreover, lipase activity consistently yielded superior loaf volumes, a reflection of enhanced emulsification capacity and more stable gas cell structures. Additional benefits include delayed staling, improved antioxidant retention, and heightened flavor complexity. Together, these data reinforce that lipase-mediated lipid remodeling not only augments sensory quality but also delivers quantifiable nutritional advantages when strategically applied during processing (Stemler & Scherf, 2023).

**Table 1:** Nutritional Benefits of the Lipase Intervention in Wheat based bakery products (2020–2025).

Parameter Enhanced	Conventional Matrix	Lipase-Enriched Matrix	Lipid Classes Modified	Lipid Class Content	Functional Impact in Bread Formulation	Reference
Loaf Volume	Moderate	Elevated	Lysophospholipids, glyceroglycolipids	–	Improved gluten-lipid interaction enhances gas retention	(Stemler & Scherf, 2023)
Nutrient Density	Standard	Elevated	Hydrolyzed lipids	–	Enhanced lipid digestibility and nutrient absorption	(Melis & Delcour, 2020)
Baking quality	Low	High	Triacylglycerols, diacylglycerols, monoacylglycerols, glycerophosphocholine and lysoglycerophosphocholine	0.2 mg/g to 186.5 mg/g	Monoacylglycerols act as natural emulsifiers	(Stemler et al., 2025)
Texture and Shelf-Life	Basic	Improved	Flavor-active lipid derivatives, starch–lipid complexes	↑ Specific volume, ↓ Hardness, ↓ Chewiness	Enzymatic action led to uniform starch matrix, increased loaf volume, reduced gumminess and improved structure	(Coţovanu & Mironeasa, 2022)
Shelf-Life Stability	Short	Extended	Reduced lipid oxidation products	↓ TBARS levels	Lower staling and oxidative rancidity	(Melis & Delcour, 2020)
Emulsification	Weak	Strong	Mono- & Diacylglycerols	↑ Surface-active forms	Lipase-generated polar lipids enhanced in-situ emulsification, dough stability, and reduced staling—replacing synthetic emulsifiers	(Stemler & Scherf, 2023)
Enzyme Traceability & Regulation	Unverified enzyme presence	Quantifiable enzyme inclusion	Lipase	<20 ppm detectable	Standardized LC–MS/MS proteomics enables detection and quantification of lipase FE-01 in wheat flour and bread for regulatory compliance	(Luo et al., 2021)
Digestibility	Moderate	Enhanced	Lipid hydrolysis products	↑ Free fatty acids	Improved bioavailability of nutrients	(Chen, Mense, et al., 2024)

### 4.3 Bound lipids and essential fatty acids

Lipases have been effectively employed as catalysts in food processing to selectively hydrolyze fats and oils, releasing fatty acids into food items. Lipases produce monoglycerides and free fatty acids by breaking down the ester bonds of glycerides and fatty acids at triglyceride sites 1 and 3, increasing the amount of polar lipids in the dough (Park & Park, 2022). Additionally, it strengthened the gluten net, which increased the volume of the bread loaf. The materials strengthen the gluten network by binding with protein. According to one researcher, fatty acids that make up lipids interact with starch granules by forming complexes between the nonpolar regions of lipids and starch after non-covalent bonding. The development of amylose-lipid complexes enhances flour stability and restricts water diffusion. Novozymes (Lipopan F) increases the bulk density of dough while decreasing surface viscosity by lowering surface tension at the air-water interface (Njus, Kelley, Tu, & Schlegel, 2020).

The employment of techniques like adding fatty acid to increase their physiological and antioxidant activities is motivated by the disadvantage of deteriorating. Consequently, lipases increase dough stability by generating glycerides with emulsifying qualities through their action on dough lipid. A method for future researchers to examine how lipase and protease work together to improve cookie compositions (Liaquat et al., 2025).

## 5 Quality improvements in bakery products

### 5.1 Improvement in loaf volume

When lipases are added, the volume of bread loaf (LV) increases, according to several authors (Melis & Delcour, 2020; Stemler & Scherf, 2023). Additionally, a well-stretched, whiter & structured crumb can be obtained with increased softness of crumb, improved pore homogeneity, and a reduced crumb pore diameter. Increased gas cell stability, whose process was previously explained with regard to changing the proteins of gluten/creating stable interfacial lipid layers, has been linked to the rise in bread LV & homogeneous crumb structure that was achieved. In this connection, the differences in the way polar and nonpolar lipids affect the stability of gas cells arise as a result of their molecular structure and their interfacial behavior. Polar lipids, such as phospholipids, PC, LPC, and galactolipids (MGDG and DGDG), contain amphiphilic headgroups that allow them to easily form and stabilize interfacial films. They create these films that support the walls of the gas cells, reducing surface tension, boosting the flexibility of the film, and underpinning the lamellar mesophase structures that are necessary for retaining the bubbles in the dough during the proofing and early baking process (Melis & Delcour, 2020; Min et al., 2020). The breakdown of these polar lipids by lipases generates lysolipids, which, in turn, are even more surface-active and add firmness to the interfacial films, hence boosting loaf volume. Conversely, non-polar wheat lipids (triacylglycerols) lack polar head groups and therefore cannot adequately adsorb to the interface, leading to disruption of the structured polar lipid monolayer, an increase in surface tension, and increased potential for film rupture, making the gas cells more susceptible to both coalescence and collapse (Stemler & Scherf, 2023). Furthermore, excess non-polar lipids water down the concentration of the functional polar lipids in the dough liquid, which in turn makes it even harder for the dough to hold onto its gas. However, due to their varying specificities, not all lipases are equally effective at improving LV (Melis & Delcour, 2020). The ‘perfect’ baking lipase exhibits optimal activity on a variety of flour substrates, including phospho- and galactolipids and (tri)acylglycerols, and produces optimal gas cell stability, enabling the replacement of often-employed surfactants. Lipases that exhibit strong activity toward polar lipids, particularly DGDG & PC, perform well in baking and also showed that lipases that hydrolyze either PC or DGDG require a larger dosage than lipases that hydrolyze both lipid types (Al-Tarifi et al., 2020). Melis and Delcour (2020) discovered that the conversion of polar lipids into their more polar lysolipids raises bread LV due to its beneficial effect on gas cell stabilization. Additionally, lipases that primarily operate on glycolipids and less so on phospholipids have been shown to be more effective in creating bread LV. Additionally, Melis and Delcour (2020) observed that bread loaf volume decreases in a lipase dose-dependent manner during overdoses. They connected this to the composition of lipids and the associated monolayers that developed at the interfaces of gas cells. Using several lipases and DATEM at different fermentation durations, a unique bread LV was produced. With the exception of Lipopan 50 in a short fermentation procedure & Lipopan Xtra in a lengthy fermentation process, they discovered that DATEM and all enzymes raise particular bread LV. In our view, this highlights once more how crucial it is to have optimal lipid conversion and breakdown in order to produce high-bread LV. Finally, evident in terms of improved loaf volume and crumb color, the synergistic effect of lipase and lipoxygenase activities on polar lipids was evident in terms of improved loaf volume and crumb color.

### 5.2 Shelf-life extension through lipid modification

Bread loses its intended crumb softness and freshness-associated scent while being stored. The term “staling” describes this occurrence, which is characterized by the firming of the crumb and the loss of flavor and crispness in the crust. Initial crumb hardness has been attributed to amylose gelation and crystallization. Long-term crumb firming is caused by amylopectin retrogradation, the creation of a partially crystalline starch network, rigid and water migration over crumb to crust, and molecules (i.e., from gluten to starch). It has been demonstrated that adding lipase affects the staling process and, consequently, lengthens the shelf life of bread (Melis & Delcour, 2020; Temkov & Mureșan, 2021). Due to increased amylose-lipid complex formation, this has been linked to the in situ creation of surfactants, which are made up of free fatty acids & monoacylglycerol and have an effect on crumb firming.

In fact, due to the steric impediment that diacylglycerol (DAGs) or phospho- or galactolipids containing two fatty acids (FAs) encounter, amylose is more readily complexed by free fatty acids/monoacylglycerol and most likely also by lysogalactolipids and lysophospholipids. Improved bread softness and springiness have been linked to this increased complex formation upon lipase addition, as compared to control bread manufacturing (Melis & Delcour, 2020). Moreover, there is no variation found in the amount of moisture diffusion from crumb to crust; they did remark on a possible antifirming impact of the Lipopan F on cake crumb in high-ratio layer cakes. Other authors also reported improved shelf life, softness, and crumb structure (Stemler et al., 2025). But when it comes to baking bread, it's possible that even if the cake crumb is initially softer, it nevertheless firms up at a rate comparable to that of cakes without lipase.

### 5.3 Antioxidant stability

Wheat has a strong antioxidant defense against DPPH and free oxygen radicals. Three assays are frequently used to evaluate antioxidant stability: DPPH (2,2-diphenyl-1-picrylhydrazyl), ABTS (2,2-azino-bis-3-ethylbenzthiazoline-6-sulphonic acid), and PCL (photochemiluminescence). The antioxidant activity of astaxanthin and its impact on lipid stability, physicochemical attributes, and sensory qualities of astaxanthin-enriched cookies. Antioxidant capacity was assessed using DPPH, hydroxyl radical scavenging, and FRAP (Ferric Reducing Antioxidant Power) assays. Lipid stability was monitored through peroxide, *p*-anisidine, and TOTOX (Total Oxidation Value) in cookies formulated with 10%, 15%, and 20% astaxanthin. Astaxanthin showed strong antioxidant activity, with 97% DPPH inhibition. Increasing astaxanthin levels significantly reduced cookie hardness without affecting taste acceptability. Storage influenced peroxide and *p*-anisidine values but not TOTOX value. These findings support the ability of astaxanthin as a functional food content with health-promoting properties (Al-Tarifi et al., 2020).

Moreover, the DPPH assay calculates the antioxidant activity by calculating the extract's percentage suppression of DPPH free radicals. Absorbance in this technique is calculated at 517 nm. By measuring absorbance at 734 nm, ABTS, on the other side, quantifies the suppression of the ABTS radicals. According to both quantitative and qualitative analyses of flavonoids and phenolic acids, wheat has the increased antioxidant stability (up to 6899 μmol/100 g). Anthocyanins, flavonols, phenolic acids & flavones are the main phenolic components present in various colored wheat that have the strongest antioxidant properties (Sanghvi, Singh, Bishoyi, & Joshi, 2025).

Incorporation of Cashew Apple Pomace Powder (CAPP) into wheat flour-based biscuits (at 5% to 15%) significantly improved nutritional and functional properties. Compared to wheat flour, CAPP contained higher protein (10.31 g/100 g), fiber (14.80 g/100 g), and antioxidant content (DPPH: 485.36 mg TEAC/100 g). Biscuits enriched with up to 10% CAPP showed enhanced oxidative stability (up to 2.79 times), mineral content, and consumer acceptability, supporting its potential as a value-added ingredient in wheat-based bakery products (Osei et al., 2025). Anthocyanin amount reports for roughly 69% of blue pigmented wheat's potential to scavenge free radicals, while phenolic acids account for **19%**. Cereals' bran layer consists of natural antioxidants and could be added to food to enhance nutrition or used as a component in functional foods (Osei et al., 2025).

## 6 Technological considerations in lipase application

### 6.1 Phenolic antioxidants

**Fig. 3:**
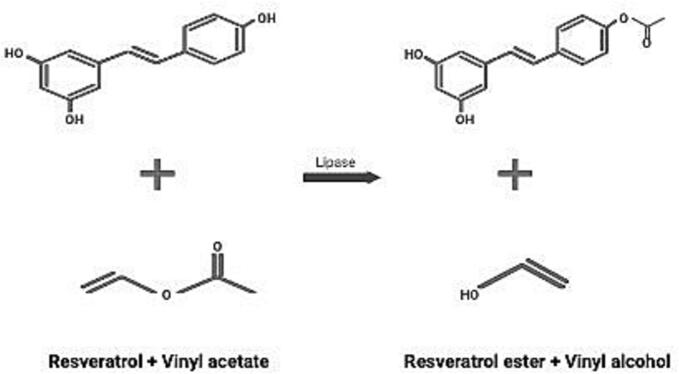
Lipase-catalyzed esterification of resveratrol with vinyl acetate demonstrating enzymatic synthesis of resveratrol esters.

Phenolic chemicals are secondary metabolites that are mostly found in fruits, vegetables, and cereals. They give these foods their color, flavor, and aroma. These substances are primarily divided into curcuminoids, stilbenes, phenolic acids, and flavonoids. They decrease adipogenesis and aid in the management of conditions such as hyperglycemia, diabetes, hypertension, obesity, and hyperlipidemia (Petropoulos, Di Gioia, Polyzos, & Tzortzakis, 2020). Although there are advantages to consuming these phenolic compounds, their low bioavailability and physiological stability limit the quantity needed to achieve their antioxidant potential. This is why phenol polymers have been synthesized using enzymes like lipases and glucanotransferases (Fig. 3) (Li et al., 2021; Nagarajan et al., 2020).

Phenolic chemicals included in food can have antioxidant properties in a variety of ways. An energetically stable species with an even number of electrons is created in the 1st when a hydrogen atom from a portion of the phenolic compound is transferred to free radicals for neutralization by a mechanism that transfers one electron from the phenolic compound, leaving it with an odd number of electrons dispersed throughout the aromatic ring. In the 2nd, the phenolic compound transfers a proton to create an anion; an electron is then donated, leading to the stabilization of free radicals. The 3rd scenario is caused by transition metal chelation (Zeb, 2020). Although, due to intermolecular interactions with the macronutrients, phenolic chemicals change the antiobesity, anticarcinogenic, antibacterial, and antioxidant properties of diets, either enhancing or decreasing them. Thus, by comprehending these modifications, it would be possible to take advantage of them to enhance the final product's quality, stability, organoleptic qualities, and shelf life (Tian, Chen, Tilley, & Li, 2021; Zhang, Cheng, Wang, & Fu, 2021).

### 6.2 Prebiotics and biosurfactants

The *gut* microbiota (probiotics) breaks down prebiotics, which are nutrients. Their connection to human health has garnered increasing attention in recent years (Abouelela & Helmy, 2024). The nutritional and bioactive qualities of food matrices could be altered by the metabolic activity of microbes during fermentation, which makes them advantageous for ingestion. The intestinal bacteria ferment some short-chain fatty acids, such as oligo- and polysaccharides, that are difficult for the intestines to digest. The progress made thus far is quite encouraging, even though additional research studies showing the complete efficiency of the novel drugs & yield-boosting techniques are needed. Amphiphilic molecules with emulsifying and biosurfactant properties and increased prebiotic activity consist of anti-inflammatory, antibacterial, and cytotoxic properties, although lipase-catalyzed esterification of prebiotic oligosaccharides and the oligosaccharide remain water soluble (Reyes-Reyes et al., 2022).

Other raw materials, like sucrose, can now be used for value-added processes like sugar fatty acid esters (SFAEs), which are compounds with a carbohydrate moiety and one or more fatty acids because they are lipophilic and non-ionic surfactants that could be made with lipases within a single-step enzymatic reaction thanks to the acylation process (Dini, Bekhit, Roohinejad, Vale, & Agyei, 2024). The food sector uses these goods due to their excellent safety and biodegradability. Both enzymatic and chemical procedures are used to synthesize them; the latter is the most current and has been researched since the reactions are carried out in softer circumstances and remove the label of synthetic chemicals, giving the consumer more confidence.

When discussing the wheat dough, biosurfactants play a significant role in the fermentation process. In situ lipase activity in the dough breaks down endogenous lipids to create the most relevant biosurfactants. During the mixing and fermentation process, lipases in the dough specifically target and break down galactolipids and phospholipids to produce lyso-galactolipids, lyso-phospholipids, monoacylglycerols (MAGs), and free fatty acids, all of which possess strong surface-active properties. The most effective action of biosurfactants is in gas cell stabilization, dough elasticity, and crumb uniformity for wheat dough, which is achieved by the emulsifying monolayers that are created at the gas-liquid interface by MAGs, lyso-galactolipids, and lyso-phospholipids, much like synthetic clean-label emulsifiers. Furthermore, MAGs and lyso-lipids contribute to the formation of amylose-lipid complexes, which slows down the retrogradation process and lengthens shelf life. The emulsifiers in clean-label products function in the same way as these compounds. The MAGs and lysolipids also form complexes with amylose, thus preventing retrogradation and extending the shelf life of baked products. Recent evidence suggests that some of the fatty acids and lysolipids produced during hydrolysis can stimulate the growth of beneficial microorganisms, supporting a prebiotic effect when consuming lipase-treated doughs (Reyes-Reyes et al., 2022).

### 6.3 Lipase substrates in bread making

A common bread formula contains lipids from wheat flour, bakery shortening/surfactants & margarine. Lipids of endogenous wheat flour constitute 2.0% to 2.5% of flour by weight, depending on genetic and environmental factors and milling and lipid extraction methods. Despite such low quantities, lipids from wheat flour are important in breadmaking. They are split into starch-associated lipids (40%), which are found in the non-starch lipid & starch granule. The latter are further classified into bound and free lipids by organic solvent extractability. After extracting free lipids with hexane, bound lipids are removed with water-saturated butanol. This extraction method does not distinguish between classes; it offers information regarding their interaction/entrapment in other flour components such as proteins of gluten (Melis & Delcour, 2020).

Phospholipids like phosphatidylcholine (PC) and galactolipids like digalactosyldiacylglycerol (DGDGs) are non-starch polar lipids. They mostly come from amyloplast membrane remnants. Lysophosphatidylcholine (LPC), lysophosphatidylethanolamine (LPE), and lysophosphatidylglycerol (LPG) dominate starch internal lipids. Linoleic (60%) and palmitic (20%) are the most abundant FAs in wheat. Shortening or margarine are bakery fats (Jin, Zheng, Zhang, Gao, & Yu, 2020; Luo et al., 2021; Melis & Delcour, 2020; Zhao & Xu, 2022). Margarine includes up to 20% non-lipid material, while shortening contains nearly 100% lipids. In shortening and margarine, TAGs are dominant. Bakery fat is usually added at 2%–4% of flour weight. SSL (Sodium Stearoyl Lactylate), MAGs, and DATEM are common non-essential bread additives at up to 0.5% of flour weight. They lack a hydrolysable ester link; therefore, lipases cannot utilize them except MAGs. By lowering their surface tension, surfactants are amphiphilic molecules that stabilize and emigrate to liquid/liquid or gas/liquid interfaces. Emulsifiers are surfactants that work at the interface of immiscible liquids like water and oil. Most gas-liquid interfaces are stabilized in fermented wheat flour dough during bread manufacturing. Dough is a complicated semi-solid foam, or gas bubble separation in liquid. For the sake of this debate, “surfactant” is used instead of “emulsifier.” The latter will be used to describe some components' emulsifier functionality. Wheat endogenous lipases are admissible to bread manufacturing, although to varying degrees (Stemler & Scherf, 2023).

Whole wheat flour is prone to FA rancidification due to endogenous lipase activity. High temperatures and long storage times promote enzymatic rancidification. Rancidification reduces flour functioning, nutritional quality, and sensory appeal, affecting end uses. The lipase activity in white flour is moderate, which reduces the hydrolysis of endogenous lipids and fatty acid rancidification. The majority of lipase activity is detected in the germ and bran fraction. Note that lipase activity in wholegrain flour can hydrolyze native wheat lipids and bakery fat (Al-Tarifi et al., 2020).

### 6.4 Impact of lipid hydrolysis products on bread volume

Recently, lipases have been applied in the formulation of cakes and bread, in comparison to amylase and xylanases. This is likely due to their broad specificity, which creates uncertainty about which of their lipolytic activities is advantageous or disadvantageous depending on the kind of wheat lipid hydrolyzed (Melis & Delcour, 2020).

Accompanied by the lipase, lipolysis is a hydrolysis reaction that breaks down ester/fat into their alcohol/glycerol and fatty acid. 190 Lipase-catalyzed hydrolysis is superior to traditional methods, such as producing fatty acids among unstable oil that contains unsaturated fatty acids and conjugated, in which high temperatures and pressures cause harmful lipid oxidation (Melis & Delcour, 2020).

However, enzymes that hydrolyze galactolipids and phospholipids, except for sn-1 MAGs or TAGs, appear to (partially) solve this issue (Kergomard et al., 2022). When adding lipases, it's also significant that a high concentration of sucrose could block or delay the processes of other hydrolytic enzymes, like xylanases and amylases.

## 7 Safety, regulatory, and consumer acceptance

The application of lipases in wheat-based bakery products is supported by robust safety evaluations and regulatory approvals. In 2025, Health Canada authorized the usage of lipase derived from *Komagataella phaffii* LALL-LI2 in bread, white flour, whole wheat flour, and unstandardized bakery products, following a comprehensive premarket assessment that included allergenicity, toxicology, and dietary exposure (*Notice of modification to the List of Permitted Food Enzymes to enable the use of lipase from Komagataella phaffii LALL-LI2 in bread, flour, whole wheat flour and unstandardized bakery products*, 2025). The enzyme was approved for use under Good Manufacturing Practice (GMP) conditions, aligning with international standards set by JECFA and Codex Alimentarius. From a technological standpoint, lipases are considered processing aids in many jurisdictions, including the EU and North America, and are typically inactivated during baking. This exempts them from mandatory labeling, although this practice has raised consumer transparency concerns in clean-label markets (Stemler & Scherf, 2023). Nonetheless, lipases are increasingly accepted by consumers as natural alternatives to synthetic emulsifiers like DATEM, especially when used to improve dough machinability, loaf volume, and shelf life in wheat-based systems.

Recent studies using wheat flour type 405 have demonstrated that lipases can significantly reduce batter stickiness and improve rheological properties in cakes and breads, further validating their functional and safe use in wheat-based matrices (Osei et al., 2025; Stemler et al., 2025). As enzyme biotechnology advances, maintaining traceability, allergen control, and regulatory harmonization will be critical to sustaining consumer trust and ensuring safe, effective implementation in bakery innovation.

### 7.1 Regulatory framework for lipase utilization in bakery applications

The Food Safety and Standards Authority of India, through the Food Safety and Standards (Food Products Standards and Food Additives) Regulations, 2011, provides a structured framework for the use of enzymes, including lipases, in food manufacturing. Lipases are categorized as either enzyme preparations or processing aids, with their application subject to strict regulatory oversight concerning source origin, permissible limits, labeling, and safety documentation (*Food Safety and Standards (Food Products Standards and Food Additives) Regulations* 2011, 2020). Major global authorities, including the EU (European Commission), the FDA (US Federal Government), and the Codex Alimentarius, provide a generally uniform regulatory structure regarding the incorporation of lipase enzymes into baked goods. Additionally, lipases used in food production in a commercially processed bakery are recognized as “processing aids,” meaning that while they have a technological purpose in dough development, they will typically not subsist heat during the baking process. European Regulation (EU) 1332/2008 mandates that food enzyme suppliers need to demonstrate safety through risk assessment, indicate a technological need for the enzyme, and prove that the enzyme does not pose a risk to consumers, and as a result, a variety of enzymes from various microorganisms have been evaluated by EFSA for use in cereal and grain products (http://data.europa.eu/eli/reg/2008/1332/oj). In addition, the FDA (Food and Drug Administration) considers various lipase enzymes to be GRAS (Generally Recognized as Safe) when produced by microorganisms under Good Manufacturing Practices (GMP), and hence, there is no labeling requirement should the final baked bread contain no residues of the lipase. Codex offers international guidance on the purity of food enzymes, assurance that no adverse effects can occur when used, and GMP compliance. Although some specific jurisdictions regulate lipases specifically by the type of enzyme, the majority of global regulatory authorities focus their efforts in the area of encapsulated safety of the source organism from which the lipase enzyme was derived, no allergenic components present in the source organisms or their derivatives, no residual enzymatic activity of lipase in the finished product, and compliance with Good Manufacturing Practices (GMP). As regulated across multiple jurisdictions, these laws give broad-based support for the safety and effectiveness of lipase enzymes as tools for maximizing dough quality and productivity.

Of all the above-mentioned regulatory systems, major emphasis is on organism safety, absence of allergens, lack of residual enzymatic activity, and compliance with GMP rather than on product-specific limitations. All these global regulatory agencies support the wide acceptance of lipases as safe and efficient dough-processing and product-quality-enhancing tools in bakeries.

## 8 Advantages of lipase-enriched bakery products

Wheat-based bakery items remain a dietary staple due to their affordability, palatability, and processing adaptability. The enzymatic application of lipases introduces novel opportunities to enhance both the nutritional and technological attributes of such products. Several studies have shown that the use of lipases increases loaf size (by 15–35%) and dough stability (by 10–20%) and prolongs crumb softening (by 10–25%), resulting in the potential for an extended shelf life (1–2 days longer), depending on the amount of lipase applied and formulation used (Melis & Delcour, 2020; Stemler et al., 2025). Lipolytic hydrolysis of monogalactosyl diacylglycerides, galactosyl diacylglycerides, and N-acyl phosphatidyl ethanolamines produces very surface-active lysolipids and monoacylglycerol, which enhance the association between gluten and lipid, leading to an increase in carbon dioxide retention, elasticity, and the production of a more finely textured homogeneous crumb structure. The enzymatic hydrolysis of lipids also improves the nutritional value of baked goods due to the release of bound fatty acids and sterols, leading to increased bioaccessibility (30–50%), improving the body's absorption of key minerals (magnesium, iron, and zinc) (Guo et al., 2020).

Chen, Yu, et al. (2024) found that lipase activity increases the production of various types of aroma-enhancing compounds (volatile esters and aldehydes) in addition to increasing the amount of lipids present when baked with flour prior to baking. Lipidomic analysis indicated that the concentration of many important aroma-enhancing compounds increased from 1.5- to 3-fold in comparison to controls (no lipids) and were capable of enhancing flavor complexity without requiring the addition of synthetic additives. Through stimulating the formation of complexes of amylose and lipids, it restricts the rate of oxidative degradation of these complexes and thus reduces the retrogradation of starch and moisture loss from baked products. Lipases can also be considered a clean-label emulsion agent, providing an all-natural alternative to DATEM or SSL. Additionally, through their interface modification, lipases provide opportunities to include wheat bran, dietary fiber, prebiotic materials, and phenolic materials into baked goods, thus offering expansion opportunities for bakeries to create nutrient-dense, functional, and consumer-preferred products.

## 9 Current challenges

Despite the growing application of lipases in enhancing the nutritional and functional qualities of wheat-based bakery products, several challenges hinder their consistent performance and broader industrial adoption. A primary issue lies in the variability of wheat flour composition, particularly in lipid and gluten content, which affects enzymatic response and dough behavior. Inconsistent effects on dough rheology, loaf volume, and crumb structure are commonly observed due to differences in endogenous lipid profiles (Melis & Delcour, 2020). Additionally, most commercial lipases exhibit limited substrate specificity and reduced activity toward complex lipids such as galactolipids and N-acyl phospholipids, which are particularly relevant in wheat matrices (Stemler & Scherf, 2023). Cake systems present further complexity, where multiple lipid types, both intrinsic and added, interact with emulsifiers and surface-active proteins under varying thermal and shear conditions. These interactions make it difficult to predict lipase behavior, highlighting the need for a deeper understanding of enzyme–lipid–protein dynamics in such environments (Stemler et al., 2025).

Technologically, the lack of real-time monitoring tools for lipid hydrolysis limits process optimization and quality control. Variations in formulation components, fat content, fermentation times, and processing conditions further complicate the standardization of lipase functionality across different wheat-based products. Moreover, regulatory ambiguity surrounding lipase classification as processing aids can hinder consumer transparency, especially in clean-label markets. This raises challenges in product labelling and acceptance, despite the benefits lipases offer. To overcome these limitations, future work should focus on advanced enzyme engineering, flour-specific formulation strategies, and the development of real-time lipidomic diagnostics. These efforts will be essential to ensure consistent performance, regulatory compliance, and consumer trust in lipase-enhanced bakery innovations.

## 10 Conclusion

The application of lipases in wheat-based bakery products represents a promising frontier in food biotechnology, enabling simultaneous enhancement of nutritional quality and product functionality. Through targeted lipid hydrolysis and modification, lipases significantly influence dough behavior, loaf volume, shelf stability, and sensory properties. These enzymatic transformations not only improve structural and organoleptic attributes but also promote health-oriented profiles by increasing the availability of essential fatty acids, bioactive lipids, and micronutrients. Moreover, lipase activity contributes to the generation of functional compounds with antioxidant and prebiotic properties, supporting clean-label formulation strategies and aligning with consumer demand for healthier and minimally processed foods. The valorization of wheat bran and incorporation of structured lipids further expand the scope of lipases in sustainable and functional food design. However, challenges remain in ensuring enzyme specificity across different flour matrices, maintaining consistent performance at an industrial scale, and adhering to regulatory and safety standards. Advances in enzyme engineering, lipidomics, and real-time process control offer avenues for optimizing lipase-based interventions and achieving more predictable outcomes. Future research should emphasize a multidisciplinary approach that integrates biochemical innovation with sensory science and market insights. Such strategies will be crucial in maximizing the commercial and nutritional potential of lipases in modern bakery systems. By bridging enzymatic science with food product innovation, lipases can serve as vital tools in developing next-generation wheat-based bakery products that are not only nutritionally enriched but also technologically superior and consumer-oriented.

## CRediT authorship contribution statement

**Hetsi Goswami:** Methodology, Data curation, Conceptualization. **Ashok Kumar Bishoyi:** Supervision, Software, Resources. **Gaurav Sanghvi:** Writing – review & editing, Writing – original draft, Validation.

## Ethical statement

This article does not contain any studies involving human participants or animals performed by any of the authors.

## Declaration of competing interest

The authors declare the following financial interests/personal relationships which may be considered as potential competing interests: Gaurav Sanghvi reports was provided by Marwadi University. Gaurav Sanghvi reports a relationship with Marwadi University that includes: employment. If there are other authors, they declare that they have no known competing financial interests or personal relationships that could have appeared to influence the work reported in this paper.

## Data Availability

No data was used for the research described in the article.

## References

[bb0005] Abd Razak N.N.B. (2022).

[bb0010] Abdelaziz A.A., Abo-Kamar A.M., Elkotb E.S., Al-Madboly L.A. (2025). Microbial lipases: Advances in production, purification, biochemical characterization, and multifaceted applications in industry and medicine. Microbial Cell Factories.

[bb0015] Abdul Manan S.F., Li J., Hsieh C.F., Faubion J., Shi Y.C. (2022). Extraction of non-starch lipid from protease-treated wheat flour. Journal of the Science of Food and Agriculture.

[bb0020] Abouelela M.E., Helmy Y.A. (2024). Next-generation probiotics as novel therapeutics for improving human health: Current trends and future perspectives. Microorganisms.

[bb0025] Akram F., Mir A.S., Haq I.U., Roohi A. (2023). An appraisal on prominent industrial and biotechnological applications of bacterial lipases. Molecular Biotechnology.

[bb0030] Al-Tarifi B.Y., Mahmood A., Assaw S., Sheikh H.I. (2020). Application of astaxanthin and its lipid stability in bakery product. Current Research in Nutrition and Food Science Journal.

[bb0035] Astrup A., Magkos F., Bier D.M., Brenna J.T., de Oliveira Otto M.C., Hill J.O., Krauss R.M. (2020). Saturated fats and health: A reassessment and proposal for food-based recommendations: JACC state-of-the-art review. Journal of the American College of Cardiology.

[bb0040] Chen Y., Yu Y., An X., Zhang H., Gong W., Liang Y., Wang J. (2024). Changes in lipid metabolites and enzyme activities of wheat flour during maturation. Foods.

[bb0045] Chen Z., Mense A.L., Brewer L.R., Shi Y.C. (2024). Wheat bran layers: Composition, structure, fractionation, and potential uses in foods. Critical Reviews in Food Science and Nutrition.

[bb0050] Coţovanu I., Mironeasa S. (2022). An evaluation of the dough rheology and bread quality of replacement wheat flour with different quinoa particle sizes. Agronomy.

[bb0055] Dai Y., Tyl C. (2021). A review on mechanistic aspects of individual versus combined uses of enzymes as clean label-friendly dough conditioners in breads. Journal of Food Science.

[bb0060] Dangi P., Chaudhary N., Paul A., Sharma A., Dutta I., Razdan R. (2023).

[bb0065] Dini S., Bekhit A.E.D.A., Roohinejad S., Vale J.M., Agyei D. (2024). The physicochemical and functional properties of biosurfactants: A review. Molecules.

[bb0070] Feng J., Xu B., Ma D., Hao Z., Jia Y., Wang C., Wang L. (2022). Metabolite identification in fresh wheat grains of different colors and the influence of heat processing on metabolites via targeted and non-targeted metabolomics. Food Research International.

[bb0075] Ferreira-Dias S., Osório N., Tecelão C. (2022). Current developments in biotechnology and bioengineering.

[bb0080] (2020). Food Safety and Standards (Food Products Standards and Food Additives) Regulations 2011.

[bb0085] Gihaz S., Bash Y., Rush I., Shahar A., Pazy Y., Fishman A. (2020). Bridges to stability: Engineering disulfide bonds towards enhanced lipase biodiesel synthesis. ChemCatChem.

[bb0090] González-Thuillier I., Pellny T.K., Tosi P., Mitchell R.A., Haslam R., Shewry P.R. (2021). Accumulation and deposition of triacylglycerols in the starchy endosperm of wheat grain. Journal of Cereal Science.

[bb0095] Guan W., Zhang D., Tan B. (2023). Effect of layered debranning processing on the proximate composition, polyphenol content, and antioxidant activity of whole grain wheat. Journal of Food Processing and Preservation.

[bb0100] Guo Y., Cai Z., Xie Y., Ma A., Zhang H., Rao P., Wang Q. (2020). Synthesis, physicochemical properties, and health aspects of structured lipids: A review. Comprehensive Reviews in Food Science and Food Safety.

[bb0105] Hamdan S.H., Maiangwa J., Ali M.S.M., Normi Y.M., Sabri S., Leow T.C. (2021). Thermostable lipases and their dynamics of improved enzymatic properties. Applied Microbiology and Biotechnology.

[bb0110] Jiang X., Zou X., Chao Z., Xu X. (2022). Preparation of human milk fat substitutes: A review. Life.

[bb0115] Jin M., Zheng W., Zhang Y., Gao B., Yu L. (2020). Lipid compositions and geographical discrimination of 94 geographically authentic wheat samples based on UPLC-MS with non-targeted lipidomic approach. Foods.

[bb0125] Kergomard J., Carrière F., Paboeuf G., Barouh N., Bourlieu-Lacanal C., Vié V. (2022). Modulation of gastric lipase adsorption onto mixed galactolipid-phospholipid films by addition of phytosterols. Colloids and Surfaces B: Biointerfaces.

[bb0130] Kongor J.E., Owusu M., Oduro-Yeboah C. (2024). Cocoa production in the 2020s: Challenges and solutions. CABI Agriculture and Bioscience.

[bb0135] Kumar A., Verma V., Dubey V.K., Srivastava A., Garg S.K., Singh V.P., Arora P.K. (2023). Industrial applications of fungal lipases: A review. Frontiers in Microbiology.

[bb0140] Li F., Ning Y., Zhang Y., Huang H., Yuan Q., Wang X., Wei W. (2025). Positional distribution of DHA in triacylglycerols: Natural sources, synthetic routes, and nutritional properties. Critical Reviews in Food Science and Nutrition.

[bb0145] Li H.M., Xu T.T., Peng Q.X., Chen Y.S., Zhou H., Lu Y.Y., Yan R.A. (2021). Enzymatic acylation of rutin with benzoic acid ester and lipophilic, antiradical, and antiproliferative properties of the acylated derivatives. Journal of Food Science.

[bb0150] Liaquat A., Ashraf H., Ahsan M., Ul-Haq I., Mugabi R., Alsulami T., Nayik G.A. (2025). Enzymatic influence on dough rheology and cookie quality: Protease and lipase as functional modifiers. International Journal of Food Properties.

[bb0155] Lim S.Y., Steiner J.M., Cridge H. (2022). Lipases: It’s not just pancreatic lipase!. American Journal of Veterinary Research.

[bb0160] Luo J., Liu L., Konik-Rose C., Tian L., Singh S., Howitt C.A., Liu Q. (2021). Down-regulation of FAD2-1 gene expression alters lysophospholipid composition in the endosperm of rice grain and influences starch properties. Foods.

[bb0165] Melis S., Delcour J.A. (2020). Impact of wheat endogenous lipids on the quality of fresh bread: Key terms, concepts, and underlying mechanisms. Comprehensive Reviews in Food Science and Food Safety.

[bb0170] Min B., Salt L., Wilde P., Kosik O., Hassall K., Przewieslik-Allen A., Shewry P.R. (2020). Genetic variation in wheat grain quality is associated with differences in the galactolipid content of flour and the gas bubble properties of dough liquor. Food Chemistry: X.

[bb0175] Mota D.A., Rajan D., Heinzl G.C., Osório N.M., Gominho J., Krause L.C., Ferreira-Dias S. (2020). Production of low-calorie structured lipids from spent coffee grounds or olive pomace crude oils catalyzed by immobilized lipase in magnetic nanoparticles. Bioresource Technology.

[bb0180] Nagarajan S., Nagarajan R., Kumar J., Salemme A., Togna A.R., Saso L., Bruno F. (2020). Antioxidant activity of synthetic polymers of phenolic compounds. Polymers.

[bb0185] Nieto-Taype M.A., Garrigós-Martínez J., Sánchez-Farrando M., Valero F., Garcia-Ortega X., Montesinos-Seguí J.L. (2020). Rationale-based selection of optimal operating strategies and gene dosage impact on recombinant protein production in Komagataella phaffii (Pichia pastoris). Microbial Biotechnology.

[bb0190] Njus D., Kelley P.M., Tu Y.J., Schlegel H.B. (2020). Ascorbic acid: The chemistry underlying its antioxidant properties. Free Radical Biology and Medicine.

[bb0195] (2025). Notice of modification to the List of Permitted Food Enzymes to enable the use of lipase from Komagataella phaffii LALL-LI2 in bread, flour, whole wheat flour and unstandardized bakery products.

[bb0200] Olagunju A.I., Ekeogu P.C., Bamisi O.C. (2020). Partial substitution of whole wheat with acha and pigeon pea flours influences rheological properties of composite flours and quality of bread. British Food Journal.

[bb0205] Olagunju A.I., Oluwajuyitan T.D., Oyeleye S.I. (2021). Multigrain bread: Dough rheology, quality characteristics, in vitro antioxidant and antidiabetic properties. Journal of Food Measurement and Characterization.

[bb0210] Osei E.D., Amotoe-Bondzie A., Laar W.S., Sarpong P., Afoakwah N.A., Harangozo L., Ivanišová E. (2025). Evaluation of nutritional, antioxidant, oxidative stability, and consumer acceptability of biscuits incorporated with cashew apple pomace powder. Journal of Food Processing and Preservation.

[bb0215] Paluzar H., Tuncay D., Aydogdu H. (2021). Production and characterization of lipase from penicillium aurantiogriseum under solid-state fermentation using sunflower pulp. Biocatalysis and Biotransformation.

[bb0220] Petropoulos S.A., Di Gioia F., Polyzos N., Tzortzakis N. (2020). Natural antioxidants, health effects and bioactive properties of wild Allium species. Current Pharmaceutical Design.

[bb0225] Reyes-Reyes A.L., Valero Barranco F., Sandoval G. (2022). Recent advances in lipases and their applications in the food and nutraceutical industry. Catalysts.

[bb0230] Saini P., Kumar N., Kumar S., Mwaurah P.W., Panghal A., Attkan A.K., Singh V. (2021). Bioactive compounds, nutritional benefits and food applications of colored wheat: A comprehensive review. Critical Reviews in Food Science and Nutrition.

[bb0235] Sanghvi G., Singh N.K., Bishoyi A.K., Joshi S.J. (2025).

[bb0240] Shimane K., Ogawa S., Yamamoto Y., Hara S. (2021). The enzymatic preparation of human milk fat substitute intermediate rich in palmitic acid at sn-2 position and low-unsaturated fatty acids at sn-1 (3) positions from palm oil substrate. Journal of Oleo Science.

[bb0245] Stemler C.D., Hoefflin K.L., Scherf K.A. (2025). Effect of seven baking lipases on the lipid class composition of three different cakes. European Food Research and Technology.

[bb0250] Stemler C.D., Scherf K.A. (2023). Lipases as cake batter improvers compared to a traditional emulsifier. LWT - Food Science and Technology.

[bb0255] Temkov M., Mureșan V. (2021). Tailoring the structure of lipids, oleogels and fat replacers by different approaches for solving the trans-fat issue—A review. Foods.

[bb0260] Tian W., Chen G., Tilley M., Li Y. (2021). Changes in phenolic profiles and antioxidant activities during the whole wheat bread-making process. Food Chemistry.

[bb0265] Zeb A. (2020). Concept, mechanism, and applications of phenolic antioxidants in foods. Journal of Food Biochemistry.

[bb0270] Zhang Q., Cheng Z., Wang Y., Fu L. (2021). Dietary protein-phenolic interactions: Characterization, biochemical-physiological consequences, and potential food applications. Critical Reviews in Food Science and Nutrition.

[bb0275] Zhang Z., Zhang S., Lee W.J., Lai O.M., Tan C.P., Wang Y. (2020). Production of structured triacylglycerol via enzymatic interesterification of medium-chain triacylglycerol and soybean oil using a pilot-scale solvent-free packed bed reactor. Journal of the American Oil Chemists’ Society.

[bb0280] Zhao W., Xu X. (2022). Involvement of non-starch lipids from endogenous wheat in the development of bread dough rancidity during frozen storage. European Journal of Lipid Science and Technology.

